# Editorial: Motile active matter

**DOI:** 10.1140/epje/s10189-021-00106-w

**Published:** 2021-08-16

**Authors:** Gerhard Gompper, Clemens Bechinger, Holger Stark, Roland G. Winkler

**Affiliations:** 1 Theoretical Physics of Living Matter, Institute of Biological Information Processing and Institute for Advanced Simulation, Forschungszentrum Jülich, 52425 Jülich, Germany; 2 Fachbereich Physik, Universität Konstanz, 78457 Konstanz, Germany; 3 Institut für Theoretische Physik, Technische Universität Berlin, 10623 Berlin-Charlottenburg, Germany

The article of Purcell [1] on “Life at low Reynolds number” in the American Journal of Physics in 1977, more than 40 years ago, has generated an enormous wave of interest—as demonstrated by more than 2500 citations (Web of Science) until today. The question, how small biological microorganisms can move in a fluid with high viscocity, so that inertia is negligible and coasting is not possible, has inspired several generations of physicists and biologists. The importance of inertia is quantified by the Reynolds number $Re= \rho v_0 L_0/ \eta$, where $\rho$ is the fluid density, $\eta$ the fluid viscosity, $v_0$ a characteristic swimmer velocity, and $L_0$ a characteristic swimmer size. The Reynolds number of microswimmers is typically far less than unity, because of their small sizes and velocities—which can be compared to human swimming in a pool, where $Re\simeq 10^4$. This clearly indicates the necessity of different swimming strategies on the microscale.

Purcell was not the first to consider the swimming of biological microorganisms, and his work was predated by that of Gray and Hancock [2], who theoretically studied the swimming of sperm cells in 1955, and by that of Berg and Anderson [3], who investigated the swimming of bacteria in 1973. However, Purcell has put the question into a more general context, and showed very generally that time-reciprocal swimming moves generate no propulsion at low Reynolds numbers—the famous “scallop theorem”.

Research in the last two decades has led to an explosion of knowledge about swimming at low Reynolds numbers. In fact, although swimming looks—and is—very tedious at low *Re*, the limit $Re \ll 1$ actually greatly simplifies the experimental and theoretical analysis, because flows are laminar, and all possible complications due to turbulence are absent.

The DPG priority program SPP1726 on “Microswimmers – from Single Particle Motion to Collective Behaviour”, which provided funding for graduate students in Germany from 2014 to 2020, has provided an enormous push of the field and its development to full bloom—not only in Germany but also beyond.

The current Topical Issue summarizes some of the results obtained by members of the SPP1726. The topics covered and the results obtained as part of the SPP 1726 are too broad and too numerous to be easily summarized here. Instead, we provide a brief overview of the essential research themes.

*Research field.* Active matter is a novel class of nonequilibrium systems composed of a large number of autonomous agents. The scale of agents ranges from nanomotors, microswimmers, and cells, to crowds of fish, birds, and humans. Unraveling, predicting, and controlling the behavior of active matter is a truly interdisciplinary endeavor at the interface of biology, chemistry, ecology, engineering, mathematics, and physics. Recent progress in experimental and simulation methods, and theoretical advances, now allow for new insights into this behavior, which should ultimately lead to the design of novel synthetic active agents and materials.

*General principles and methods.* Active systems are persistently out of equilibrium, due to the continuous energy consumption of its constituent agents. This implies the absence of equilibrium concepts like detailed balance, Gibbs ensemble and free energy, as well as time-reversal symmetry. Therefore, theories of active matter have to be constructed on the basis of symmetries—like polar or nematic shape and interactions of the agents—as well as conservation laws and dynamic rules. Agent-based standard models, such as active Brownian particles and squirmers, have emerged to account for the underlying physical mechanisms in dry and wet active matter. They are complemented by continuum field theory. Methods and techniques to analyze these models and theories range from simulations, including mesoscale hydrodynamics approaches, to field-theoretical methods and dynamic density-functional theory. Another important aspect is the development of new tools for the analysis of large amounts of experimental data.

More information about these aspects can be found in Refs. [4–12] of this Topical Issue.

*Biological nano- and microswimmers.* Evolution has provided a large diversity of biological swimmers on the microscale, such as sperm cells, bacteria, and algae. Their propulsion mechanisms and navigation strategies are tailored to their function and natural environment, ranging from living organisms to soil and the open seas. Microswimmers prototypically employ cilia or flagella for propulsion, which beat or rotate, but also periodic changes of their body shape. Understanding of the underlying principles and search strategies, e.g., chemotaxis and phototaxis, allows for their targeted manipulation and control in medicine, ecology, and a multitude of technical applications. The motion of these swimmers on the microscale naturally raises the question how small a swimmer can be to still display directed motion, maybe even on the scale of a single macromolecule.

More information about these aspects can be found in Refs. [11–17] of this Topical Issue.

*Synthetic nano- and micromachines.* Various strategies for the design of autonomous synthetic nano- and micromachines have been proposed. This includes phoresis—inhomogeneous catalysis of chemical reactions (diffusiophoresis), thermal gradients (thermophoresis)—body deformations, and biology-inspired concepts, e.g., rotating helices. Such machines provide the basis for multifunctional and highly responsive (artificial) materials, which exhibit emergent behavior and the ability to perform specific tasks in response to signals from each other and the environment. The development of novel techniques facilitates control of the locomotion of individual nano- and micromachines as well as their interactions, and the design of intelligent active materials. In potential applications, the external control of nano- and micromachines is essential. Here, light has become a prime candidate, because it can be switched on and off at will, affects the particles without delay, and can be modified in strength individually for each particle.

More information about these aspects can be found in Refs. [4, 14, 18–29] of this Topical Issue.

*Swarming and collective motion.* Active agents are able to spontaneously self-organize when present in large numbers, resulting in emergent coordinated and collective motion on various length scales. Examples range from the cytoskeleton of cells, swarming bacteria and plankton, to flocks of birds and schools of fish. The mechanisms determining the emergence and dynamics of a swarm include the shape of the agents, steric interactions, sensing, fluctuations, and environmentally mediated interactions. Novel phenomena range from motility-induced phase separation to active turbulence. In biological systems, the reaction of swarms to external signals is crucial, and is often the reason for the formation of swarms in the first place. Examples range from the reaction of bird flocks to predators to the collective motion of bottom-heavy swimmer like algae in gravitational fields. In synthetic systems, light control allows for a completely new approach, in which individual or a collection of particles are steered externally to optimally reach a target or to exhibit emergent novel collective behaviors.

More information about these aspects can be found in Refs. [5, 7, 10, 20–22, 27, 30] of this Topical Issue.
